# Decoding the microbial-local immune dialogue in liver cancer: from ecological drivers to precision therapeutics

**DOI:** 10.3389/fonc.2025.1629479

**Published:** 2025-09-19

**Authors:** Yichi Xu, Bo Wen, Nan Gao, Shu Liu

**Affiliations:** Geriatric Department of the First Affiliated Hospital of China Medical University, Shenyang, Liaoning, China

**Keywords:** hepatocellular carcinoma, gut microbiota, the gut-liver axis, local immune response, the immune microenvironment, immune modulation

## Abstract

The global incidence and mortality rates of liver cancer remain persistently high, attributable to its multifaceted etiology. Recent research has increasingly focused on the interaction between gut microbiota and the liver’s immune system. The gut-liver axis, which connects the gut and liver via the portal vein system and biliary tract, is crucial in maintaining homeostasis; however, dysbiosis can compromise the gut’s barrier function. The gut microbiota exerts influence over hepatic immune cells through various mechanisms, including alterations in microbial composition, production of metabolic products, and the presence of pathogen-associated molecular patterns. These interactions contribute to immune evasion, inflammation, and remodeling of the tumor microenvironment in liver cancer, thereby affecting the efficacy of immunotherapy. Despite these insights, the precise mechanisms underlying these interactions and their potential clinical applications remain inadequately understood. This article aims to review the mechanisms of interaction between the gut microbiota and the local immune response in liver cancer, integrating the latest research advancements. Additionally, it will explore the impact of these interactions on the tumor immune microenvironment, with the objective of providing a theoretical foundation and potential strategies for prevention and therapeutic intervention.

## 1 Introduction

The global incidence and mortality rates of hepatocellular carcinoma (HCC) are on the rise, presenting a significant public health challenge. The pathogenesis of HCC is multifaceted, involving a range of factors such as viral infections, metabolic disorders, and chronic inflammation. Typically, HCC develops through a series of pathological processes, including chronic hepatitis, liver fibrosis, and cirrhosis. Recent studies have identified metabolic diseases, particularly non-alcoholic fatty liver disease (NAFLD), as major contributors to cirrhosis and HCC among younger populations (1). Additionally, emerging research indicates that the gut microbiome can influence liver metabolism and immune responses via the gut-liver axis, thereby playing a role in the onset and progression of this malignancy (2, 3). This finding offers a novel perspective on HCC treatment, as the involvement of gut microbiota and their metabolites in modulating tumor immunity is increasingly recognized, making it an emerging area of interest in HCC therapeutics.

An increasing body of evidence indicates that the development of HCC is intricately linked to significant gut microbiota imbalances. Research demonstrates that the gut microbiota of individuals diagnosed with this cancer type frequently exhibits substantial alterations, characterized by a pronounced reduction in microbial diversity (4). In patients with HCC, gut microbiota disruption is marked by a significant drop in the Bacteroidetes to Firmicutes ratio, from 0.92 ± 0.15 to 0.48 ± 0.08 (p<0.01), and a 120-fold increase in Fusobacterium nucleatum. This is closely linked to the progression of liver cirrhosis and cancer (5). Such microbial imbalances are hypothesized to activate hepatic immune cells, thereby enhancing liver inflammation and fostering the formation of a tumor microenvironment through various mechanisms, including the production of carcinogenic substances, disruption of immune system equilibrium, modification of metabolic processes, and increased intestinal permeability. These factors collectively position dysbiosis as a potential catalyst in the pathogenesis of HCC (3, 6). For instance, metabolites generated by gut microbial imbalances, such as short-chain fatty acids (SCFAs), may influence liver immune surveillance by modulating T cell function, potentially either promoting or inhibiting the progression of this disease. This interaction may represent a critical mechanism underlying the advancement of HCC (7). Furthermore, the composition and functionality of gut microbiota are not only associated with the development of HCC, but also with the diversity and compositional alterations of the microbiota, which may serve as predictors of patient responses to immunotherapy and are closely linked to the prognosis of liver cancer (8). In certain instances, modulating the composition of gut microbiota has the potential to enhance the efficacy of immunotherapeutic interventions (9). For example, modifications to the gut microbiota structure can improve the effectiveness of immune checkpoint inhibitors (10), thereby increasing patient survival rates and providing novel treatment strategies and insights.

In conclusion, liver cancer is not solely associated with traditional risk factors. It is also intricately connected to the complex interactions with gut microbiota. This article seeks to examine recent research on the microbiota-immune dialogue in liver cancer, with a comprehensive analysis of its molecular mechanisms and potential clinical applications. Such an exploration not only elucidates the pathological mechanisms underlying liver cancer but also identifies novel targets and directions for future Precision Therapeutics.

## 2 The liver: a unique nexus of immunity and microbial crosstalk

### 2.1 Distinct characteristics of the liver microenvironment

#### 2.1.1 Mechanism of immune tolerance

The liver’s immune tolerance is primarily attributed to its unique cellular composition. Kupffer cells, the liver’s resident macrophages, play a dual role in maintaining immune tolerance by both inducing Treg differentiation through PD-L1 expression (MFI = 285 ± 35) and IL-10 secretion (1200 ± 150 pg/mL), and clearing gut-derived antigens (11, 12). Notably, intestinal microbiota critically modulates this Treg-inducing capacity of Kupffer cells. Clinical studies show that gut microbiota perturbations (e.g., reduced diversity with Shannon index dropping from 3.8 to 2.9 in HCC patients) correlate with impaired Treg function and disrupted hepatic tolerance (13). Liver sinusoidal endothelial cells (LSECs) possess a unique fenestrated structure that enables them to efficiently capture antigens from the bloodstream, exhibiting an antigen-presenting capability that is 8–10 times greater than that of conventional endothelial cells (14). Through the cross-presentation of antigens, LSECs induce tolerance in CD8+ T cells (15). Furthermore, the gut microbiota plays a pivotal role in the maintenance of liver immune tolerance. It achieves this by regulating the gut-liver axis, modulating Treg function, preserving the tolerant state of Kupffer cells, and preventing the excessive activation of natural killer T (NKT) cells (16). Disruption of the intestinal barrier may permit the translocation of gut microbiota and their metabolites into the liver, thereby directly initiating liver immune tolerance.

Clinical evidence reveals three key microbiota-derived metabolites regulating liver tolerance. 1.Secondary bile acids: Elevated DCA (2.8-fold increase in HCC) activates TGR5 on Kupffer cells, increasing IL-10 production by 40% but simultaneously inducing PD-L1 expression (17). 2.Tryptophan metabolites: Kynurenine/AhR signaling upregulates hepatic PD-L1 while suppressing CD8+ T cell IFN-γ production by 65 ± 8% (18). 3.SCFAs: Butyrate (optimal 2-4mM) maintains Treg function via GPR43, but concentrations <1mM (as in HCC) reduce Foxp3+ cells by 35 ± 5% (19).

#### 2.1.2 Bidirectional communication in the gut-liver axis

The gut-liver axis functions as a bidirectional regulatory system that connects the intestine and liver, playing a crucial role in maintaining the body’s immune homeostasis through various mechanisms. The primary anatomical foundation of this axis is formed by the portal vein and bile acid circulation, while metabolite diffusion serves as a key communication pathway. Bacterial translocation and immune surveillance contribute to establishing a defense barrier. The integrity of the intestinal barrier is essential for preserving this balance. Clinical studies demonstrate intestinal barrier dysfunction as a pivotal event in HCC progression. The diminution of intestinal tight junction integrity results in the formation of leaky gut, facilitating the translocation of various microbial metabolites from the intestine into the bloodstream, which subsequently elicits an immune response. For instance, a 60% reduction in occludin, a tight junction protein, results in the detection of 10^3 copies/mL of bacterial 16S rRNA in portal vein blood (20). Elevated portal SCFA levels (butyrate +220%) paradoxically indicate barrier breach (21), while reduction in occludin correlates with increase in hepatic IL-6.

Kupffer cells efficiently eliminate these foreign substances in a CD14-dependent manner; however, persistent activation of the Toll-like receptor 4 (TLR4)/MyD88 pathway may lead to pathological fibrosis (22). In patients with chronic liver diseases, such as liver cancer, alterations in the gut microbiota are closely associated with the severity of liver disease, with reduced microbial diversity being linked to increased liver inflammation (23).

### 2.2 The evolution of microbiome-immune dynamics in liver cancer development

In the initial phases of liver cancer, dysbiosis of the gut microbiome plays a pivotal role in inducing liver inflammation and fibrosis via the lipopolysaccharide (LPS)-TLR4 axis. Empirical studies have demonstrated that the concentration of LPS in the portal vein blood of patients with liver cirrhosis is markedly elevated compared to that in healthy individuals. These LPS molecules activate TLR4 receptors on Kupffer cells, thereby initiating the NF-κB signaling pathway. This activation leads to the secretion of inflammatory mediators, such as interleukin-6 (IL-6) and senescence-associated secretory phenotype (SASP) factors, which collectively contribute to liver fibrosis and the progression to HCC (24). As liver cancer advances, the tumor microenvironment increasingly suppresses immune responses. This suppression facilitates the proliferation of immunosuppressive cells, including myeloid-derived suppressor cells (MDSCs) (25), and results in the upregulation of T cell exhaustion markers such as programmed cell death protein 1 (PD-1), along with increased co-expression of TIM-3 and LAG-3. These changes collectively establish an immunosuppressive microenvironment that enables tumors to evade immune surveillance. During the metastatic phase of liver cancer, Fusobacterium nucleatum activates the β-catenin signaling pathway in hepatic cells through the FadA adhesin, thereby facilitating tumor cell invasion (26). Concurrently, microbial metabolites, including trimethylamine N-oxide, can stimulate the MAPK pathway, leading to increased VEGF secretion and promoting tumor proliferation and migration (27).

## 3 Mechanisms of gut microbiota-driven immune dysregulation

### 3.1 Primary mechanisms of gut microbiota-driven immune regulation

#### 3.1.1 Microbial metabolites as immune modulators

The primary metabolic products of the gut microbiota encompass SCFAs, secondary bile acids (including deoxycholic acid [DCA] and lithocholic acid [LCA]), trimethylamine N-oxide, tryptophan metabolites, and LPS, among others. These metabolites not only contribute to energy metabolism but also serve as pivotal regulators of the immune response within the gut-liver axis. They modulate immune responses through various pathways, exhibiting both pro-carcinogenic and anti-carcinogenic properties (28, 29). Collectively, these metabolites interact in multifaceted ways to maintain immune homeostasis, thereby influencing the onset and progression of diseases such as liver cancer. The mechanisms through which gut microbiota metabolites exert their effects on the gut-liver axis are intricate and varied, suggesting potential novel targets for future liver disease treatments. Further investigation is warranted to elucidate the specific mechanisms by which these metabolites impact liver health and disease, with the objective of developing innovative therapeutic strategies centered on gut microbiota metabolites.

#### 3.1.2 Pathogen-mimicry: pathogen-associated molecular patterns and immune activation

Pathogen mimicry refers to the phenomenon whereby pathogens emulate host biomolecules through their molecular structures, thereby evading the host’s immune response. This mechanism is pivotal in the pathogenesis of various infectious diseases and is particularly significant in the progression of malignancies such as liver cancer, with substantial clinical implications (30). PAMPs are integral to immune activation, as they are recognized by pattern recognition receptors (PRRs), which subsequently initiate intracellular signaling pathways. These pathways lead to the production of pro-inflammatory molecules, thereby instigating the host’s initial response to infection and facilitating the subsequent activation of adaptive immunity (31). Nevertheless, pathogens can modulate the host’s immune response by mimicking host molecules, enabling their survival and replication within the host. A comprehensive understanding of the interplay between PAMPs, pathogen mimicry, and immune activation not only enhances our insight into the mechanisms underlying infectious diseases and tumorigenesis but also identifies potential targets for the development of novel vaccines and therapeutic interventions.

#### 3.1.3 Direct microbial-immune cell crosstalk

Recent studies on liver cancer have increasingly elucidated a direct communication process between microbes and immune cells, which is essential for the maintenance of immune homeostasis. Gut microbes can modulate immune cell function through direct interactions or by releasing metabolic products (32). Furthermore, these microbes influence immune responses by regulating the differentiation and functionality of immune cells. The interactions between microbes and immune cells also encompass the regulation of various cytokines and signaling pathways (33). Cytokines, as critical mediators of intercellular communication, play a pivotal role in the interaction between microbes and immune cells; they are instrumental in regulating the proliferation, differentiation, and function of immune cells, thereby shaping the intensity and nature of immune responses.

### 3.2 Multifaceted interactions between gut microbiota and HCC pathogenesis

#### 3.2.1 Disruption of the gut barrier and immune activation in liver cancer

The integrity of the gut barrier is essential for maintaining intestinal homeostasis and immune equilibrium. Its disruption is intricately linked to immune activation in liver cancer (34). A compromised gut barrier allows for the translocation of gut microbes and their metabolites into the bloodstream, facilitating the movement of PAMPs to the liver. This process modulates hepatic immune responses through mechanisms such as immune cell activation and systemic inflammation. The gut-liver immune axis maintains tolerance through balanced microbiota composition and intact intestinal barrier. Increased intestinal permeability (evidenced by 60% occludin reduction) allows microbial translocation (10^3 copies/mL bacterial 16S rRNA in portal blood), which dysregulates hepatic immune tolerance via two mechanisms: 1.Direct modulation of Kupffer cell function: LPS from translocated bacteria activates TLR4/NF-κB pathway in Kupffer cells, shifting their phenotype from IL-10-producing tolerogenic to pro-inflammatory. 2.Treg modulation: Microbial metabolites like butyrate (at physiological 2-4mM) maintain Treg suppressive function via HDAC inhibition, while dysbiosis-induced reduction (to <1mM in HCC) impairs Foxp3+ Treg stability. A reduction in the expression of the tight junction protein occludin in the gut permits bacterial products, such as LPS, to access the liver via the portal vein. The LPS-TLR4 axis is crucial in the pathogenesis of leaky gut syndrome. The translocation of LPS, resulting from increased intestinal permeability, activates TLR4 on hepatic Kupffer cells, thereby initiating the NF-κB signaling pathway and promoting the release of inflammatory mediators (35). LPS, in conjunction with signaling molecules such as adenosine triphosphate (ATP), can activate the NLRP3 inflammasome, thereby facilitating the maturation of interleukin-1β (IL-1β) and exacerbating hepatic inflammation (36). Antigens originating from the gut are transported to the liver by dendritic cells, which subsequently activate CD4+ T cells, including T helper 17 (Th17) cells, and CD8+ T cells, thereby enhancing specific immune responses (37). Pro-inflammatory factors derived from the gut, such as transforming growth factor-beta (TGF-β), can activate hepatic stellate cells, resulting in collagen deposition and fibrosis.

A randomized double-blind controlled trial (RCT) focusing on irritable bowel syndrome (IBS) demonstrated that a composite probiotic formulation containing *Lactobacillus* and *Bifidobacterium* significantly decreased intestinal permeability in IBS patients (38). Additional studies have suggested that supplementation with probiotics can reduce serum LPS levels in patients with NAFLD, thereby mitigating intestinal leakage (39). Nonetheless, the impact of these probiotics on gut barrier function in patients with liver cancer remains unclear, necessitating further investigation.

The initial activation of Kupffer cells in the liver is mediated through TLRs recognizing LPS. Kupffer cells, the primary resident macrophages in the liver, are adept at recognizing and responding to gut-derived bacterial components via various PRRs, with a particular emphasis on TLRs. Empirical studies have demonstrated a significant correlation between the activation of Kupffer cells and the pathogenesis of liver cancer, particularly under conditions of chronic inflammation. Upon recognition of LPS via TLRs, Kupffer cells trigger a cascade of signaling pathways, notably the NF-κB and MAPK pathways, which culminate in the secretion of pro-inflammatory cytokines such as tumor necrosis factor-alpha (TNF-α) and IL-6, thereby intensifying hepatic inflammation. Furthermore, LPS stimulation results in an elevated expression of hypoxia-inducible factor 1-alpha (HIF-1α) in Kupffer cells, indicating its potential role in facilitating adaptation to hypoxic conditions and modulating metabolic processes (40). In the pathophysiological development of liver cancer, Kupffer cells play a dual role by not only participating in the inflammatory response but also modulating hepatocyte proliferation and fibrosis through the secretion of various cytokines and chemokines, thereby contributing to the progression of liver cancer to a certain degree (41). The initial immune activation mechanism of Kupffer cells, which involves the recognition of LPS through TLRs, constitutes a fundamental aspect of the liver’s immune response. This mechanism also provides a critical framework for understanding liver inflammation and regeneration. Future research should focus on elucidating strategies to enhance the prognosis of liver-related diseases by regulating the functionality of Kupffer cells, thereby offering novel insights for precision treatment approaches.

#### 3.2.2 Dual immunoregulatory role of microbial metabolites

The involvement of SCFAs in the pathogenesis and progression of HCC constitutes a complex and multifaceted area of research. This field encompasses various mechanisms, including immune regulation, metabolic intervention, and the protection of the intestinal barrier. In the context of hepatic innate immune cells, SCFAs, particularly butyrate, exert regulatory effects on the polarization and functionality of liver macrophages through multiple mechanisms. Hu et al. demonstrated that gut-derived SCFAs can attenuate hepatic inflammatory responses by promoting the polarization of macrophages towards the M2 phenotype, thereby mitigating liver damage. Within the milieu of liver cancer, an optimal concentration of SCFAs is crucial for maintaining macrophage activity against tumors; however, a reduction in SCFA levels is associated with an increase in pro-tumorigenic M2 macrophages (42). Furthermore, SCFAs modulate inflammatory gene expression at the epigenetic level by inhibiting histone deacetylase (HDAC) activity, which influences their function within the liver cancer microenvironment (43). Recent investigations have revealed that butyrate modulates the activation of the NLRP3 inflammasome in liver macrophages via G protein-coupled receptors GPR43 and GPR109A. This modulation plays a dual role in the progression of liver cancer: initially suppressing excessive inflammatory responses and subsequently potentially facilitating immune suppression (44). Furthermore, research conducted by Ma et al. indicates that dysbiosis results in diminished SCFA levels, which are associated with a decrease in liver NKT cell populations and compromised immune surveillance. SCFAs have the capacity to modulate NKT cells through the CXCL16-CXCR6 signaling pathway, thereby facilitating the progression of liver cancer (4). Additionally, Hu et al. identified that gut-derived SCFAs augment the anti-liver cancer efficacy of type 3 innate lymphoid cells (ILC3s) by enhancing IFN-γ production. SCFAs not only exert a direct influence on the functionality of ILC3s but also regulate their interaction with NKT cells, collectively contributing to immune surveillance in HCC. Moreover, SCFAs impact the maturation and functionality of liver DCs. Zheng et al. demonstrated that butyrate induces the transformation of liver DCs into a tolerogenic phenotype via the GPR109A signaling pathway, thereby reducing their secretion of pro-inflammatory cytokines and altering their antigen presentation capabilities (45). Within the liver cancer microenvironment, fluctuations in SCFA levels can modulate the capacity of DCs to activate T cells, consequently affecting the efficacy of anti-tumor immune responses.

Regarding hepatic adaptive immune cells, SCFAs particularly butyrate, can enhance the anti-tumor activity of CD8+ T cells through various mechanisms, including metabolic reprogramming, epigenetic regulation, and immune checkpoint modulation. Specifically, butyrate facilitates the transition of CD8+ T cells from glycolysis to oxidative phosphorylation, thereby improving mitochondrial function and promoting T cell persistence and memory formation (46). Additionally, SCFAs can upregulate the expression of effector and memory-associated genes, such as IFN-γ, Granzyme B, and T-bet, by inhibiting HDACs, which in turn enhances the cytotoxic function of CD8+ T cells (47). Furthermore, butyrate has been shown to inhibit HDAC3 activity by 78 ± 5% at a concentration of 10 μM, while propionate reduces IL-1β secretion from Kupffer cells from 1250 ± 150 to 380 ± 50 pg/mL via the GPR43 receptor (48). SCFAs have been shown to downregulate the expression of PD-1 in CD8+ T cells within the liver cancer microenvironment, thereby mitigating T cell exhaustion and enhancing the efficacy of anti-PD-1/PD-L1 immunotherapy. Furthermore, SCFAs play a significant role in the differentiation and functional modulation of CD4+ T cell subsets. Notably, SCFAs, particularly butyrate, have been found to enhance the expression of Foxp3 and facilitate the differentiation and stability of Tregs by promoting histone acetylation in the promoter region of the Foxp3 gene (49). In the context of the liver cancer microenvironment, an optimal level of Tregs is crucial for regulating excessive inflammatory responses; however, an overabundance of Tregs may lead to immune suppression and contribute to tumor progression. The effects of SCFAs on Th17 cells are more complex; while SCFAs can inhibit Th17 cell differentiation and thereby reduce inflammatory responses, under certain conditions, they may also enhance Th17 cell functionality, thereby participating in anti-tumor immunity (50).

In the context of immune-suppressive cells, SCFAs have been shown to attenuate the recruitment of MDSCs by downregulating the expression of chemokines such as CCL2 within the liver cancer microenvironment (51). Additionally, SCFAs diminish the capacity of MDSCs to produce immunosuppressive molecules, including arginase 1 (Arg1) and inducible nitric oxide synthase (iNOS), via the GPR43 signaling pathway, thereby mitigating their suppression of T cells and NK cells. SCFAs also facilitate the differentiation of MDSCs into M1 macrophages, thereby converting them into tumor-fighting cells. Research conducted by Bi et al. has demonstrated that SCFAs modulate the function of neutrophils in the liver cancer microenvironment by influencing their recruitment and polarization (52). Specifically, SCFAs decrease the proportion of N2 (pro-tumor) neutrophils while increasing N1 (anti-tumor) neutrophils, which is advantageous for the immune-mediated control of liver cancer. Given the dual regulatory role of SCFAs, future investigations should aim to optimize their dosage and administration strategies to enhance their anticancer efficacy while minimizing potential adverse effects.

Secondary bile acids can promote the establishment of an immunosuppressive microenvironment in the liver through the activation of the TGR5 and FXR receptor pathways, consequently influencing tumor immune surveillance and evasion. Secondary bile acids, particularly elevated levels of DCA, have been shown to induce the polarization of Kupffer cells towards the M2 phenotype, which is immunosuppressive, via the TGR5 and FXR receptor signaling pathways. This polarization results in the secretion of inhibitory cytokines, including IL-10 and TGF-β, thereby suppressing anti-tumor immune responses (53). Furthermore, DCA modulates the inflammatory response of Kupffer cells through the NF-κB and STAT3 signaling pathways, promoting inflammation in the early stages of liver cancer and potentially facilitating immune suppression in later stages. The regulation of NK and NKT cells by secondary bile acids is crucial within the liver cancer immune microenvironment. Research conducted by Ma et al. demonstrated that dysregulated bile acid metabolism results in increased DCA levels, which inhibit the expression of CXCL16 in the liver, thereby reducing the recruitment of CXCR6+ NKT cells and diminishing the liver’s immune surveillance against tumors (43). Additionally, DCA impairs the cytotoxic function of NK cells against liver cancer cells by altering the expression of activation receptors and the release of cytotoxic molecules, such as perforin and granzyme B (54). Elevated concentrations of secondary bile acids have been shown to inhibit the secretion of IFN-γ by CD8+ T cells, while simultaneously promoting the proliferation of MDSCs, inducing apoptosis and functional exhaustion in CD8+ T cells, and upregulating inhibitory receptors such as PD-1 and TIM-3. These effects collectively diminish the anti-tumor efficacy of CD8+ T cells. Furthermore, secondary bile acids facilitate the differentiation of Tregs and suppress the function of Th17 cells via the FXR and TGR5 signaling pathways, thereby establishing an immunosuppressive milieu within the liver cancer microenvironment. Elevated levels of DCA have been implicated in the increased accumulation of MDSCs in the liver through the COX2-PGE2 pathway, thereby augmenting their immunosuppressive capabilities (55). Some studies propose that the strategic use of secondary bile acids in conjunction with liver cancer immunotherapy could represent a promising therapeutic avenue. Specifically, modulating the metabolism of secondary bile acids may restore the anti-tumor function of CD8+ T cells, thereby enhancing the efficacy of immunotherapeutic interventions.

Various metabolites, including TMAO, have been implicated in exacerbating liver inflammatory responses. TMAO achieves this by activating the NLRP3 inflammasome, which in turn stimulates Kupffer cells to secrete pro-inflammatory cytokines such as IL-1β and IL-18. Additionally, TMAO facilitates macrophage polarization towards the M1 phenotype through the NF-κB signaling pathway, potentially exerting anti-tumor effects during the early stages of liver cancer. However, prolonged inflammation may contribute to the progression of liver cancer (56). TMAO also influences T cell differentiation and function by promoting Th17 cell differentiation while inhibiting Treg cell function, thereby intensifying liver inflammatory responses. Furthermore, TMAO can promote the expansion and activation of MDSCs, enhancing their immunosuppressive capabilities and facilitating liver cancer progression. In parallel, tryptophan metabolites significantly impact T cell differentiation via the aryl hydrocarbon receptor (AhR) signaling pathway (57). Specifically, kynurenine has been shown to promote tumor cell expression of PD-L1 through the AhR-NF-κB pathway.

#### 3.2.3 Microbiota-mediated metabolic-immune crosstalk

In the pathogenesis of liver cancer, the gut microbiota is pivotal in facilitating the interaction between metabolic processes and immune responses. While there is presently insufficient research to establish a direct causal link between metabolic disorders induced by abnormal microbiota and the development of HCC, it is well-established that an imbalance in gut microbiota can result in metabolic abnormalities. These abnormalities are associated with the onset and progression of NAFLD, which is a significant risk factor for HCC. Furthermore, small intestinal bacterial overgrowth and dysbiosis of gut microbiota are recognized as critical factors in the progression of NAFLD to non-alcoholic steatohepatitis (NASH) and decompensated liver disease (58). Dysbiosis can influence tryptophan metabolism, which may promote liver cancer through the upregulation of sterol regulatory element-binding protein 2 (SREBP2) (59). Tryptophan metabolites are capable of modulating the expression of PD-1 via the AhR pathway, consequently impacting T cell functionality. As previously discussed, metabolites derived from the gut microbiota, including short-chain fatty acids, bile acids, and indole, exert significant effects on hepatic immune and metabolic functions through the gut-liver axis. For example, succinate can enhance the infiltration of cytotoxic T-lymphocyte-associated protein 4 (CTLA-4) positive T cells by interacting with its receptor SUCNR1, thus modifying the immune milieu of the tumor microenvironment (60). The potential implications of this research for immunotherapy in liver cancer are significant. Alterations in the liver microbiota composition may reduce the liver’s metabolic capacity for drugs, thereby influencing the effectiveness of liver cancer treatments. Empirical evidence suggests that modulating the gut microbiota can enhance the efficacy of immunotherapy for liver cancer. Interventions such as probiotics, prebiotics, antibiotics, and fecal microbiota transplantation may emerge as crucial adjuncts to liver cancer immunotherapy. These strategies enhance immunotherapy by modulating the host immune system, minimizing adverse effects, and improving survival rates among liver cancer patients.

#### 3.2.4 Microbiota-driven mechanisms of immune evasion

Recent investigations have elucidated the role of gut microbiota in modulating immune evasion mechanisms in liver cancer through multiple pathways. Primarily, gut microbiota can facilitate immune evasion by modulating the host immune system. Dysbiosis within the gut microbiota can precipitate immune system disorders, thereby impairing the efficacy of anti-tumor immune responses. For instance, metabolites produced by specific gut bacteria have been shown to inhibit immune cell activity, consequently diminishing their capacity to identify and eliminate tumor cells. Furthermore, the gut microbiota can influence immune evasion by modulating immune cells within the liver cancer microenvironment, such as by altering the activity of T cells and natural killer cells, which in turn promotes tumor growth and metastasis. Additionally, the gut microbiota may contribute to immune evasion in liver cancer by regulating the expression of immune checkpoint molecules. Empirical evidence suggests that gut microbiota can modulate the expression of PD-L1 via its metabolites, thereby suppressing anti-tumor immune responses and facilitating tumor immune evasion (61). The mechanisms of immune evasion facilitated by microbiota present significant challenges in the treatment of liver cancer.

## 4 Therapeutic potential of microbiota modulation in HCC

### 4.1 Dysbiosis in HCC patients

Dysbiosis is frequently observed in individuals with HCC. A comparative study involving HCC patients, individuals with cirrhosis, and healthy controls identified two bacterial species, *Odoribacter* sp*lanchnicus* and *Ruminococcus bicirculans*, as being significantly associated with HCC. Additionally, the study highlighted five metabolites—ouabain, taurochenodeoxycholic acid, glycochenodeoxycholate, theophylline, and xanthine—as key biomarkers (62). Research into the gut microbiota of HCC patients reveals a marked reduction in microbial diversity. Beneficial bacteria, such as *Lactobacilli* and *Bifidobacteria*, are present at diminished levels, whereas pathogenic bacteria, including *Proteus* and *Escherichia coli*, are found in increased abundance. Clinical data indicate a decrease in the *Bacteroidetes* to *Firmicutes* ratio from 0.92 in healthy individuals to 0.48 in HCC patients, alongside a notable increase in the abundance of *Fusobacterium nucleatum* from 0.01% in healthy subjects to 1.2% in HCC patients (63). Further investigations suggest that an elevated *Firmicutes* population coupled with reduced *Bacteroidetes* may contribute to immune suppression within the hepatic microenvironment. The abundance of *Fusobacterium nucleatum* is strongly correlated with the prevalence of Tregs, as evidenced by a correlation coefficient of r=0.62 (p<0.001) (64). *Fusobacterium nucleatum* facilitates the proliferation of Tregs and attenuates the activity of effector T cells by modulating cytokine levels within the tumor microenvironment and enhancing the expression of PD-L1 on the surfaces of tumor cells (65). This interaction consequently confers a survival advantage to hepatocellular carcinoma cells.

### 4.2 Microbiota typing-guided immunotherapy stratification

Microbiota typing is emerging as a novel biomarker with significant implications for immunotherapy, paving the way for the advancement of personalized medicine. *Akkermansia muciniphila*, a prevalent Gram-negative bacterium within the gut microbiome, has been identified in recent studies as having a positive correlation with patient responses to anti-PD-1 therapy (66). Patients exhibiting higher levels of *Akkermansia muciniphila* tend to experience improved survival rates and extended periods without disease progression, indicating its potential utility as a predictive biomarker for immunotherapy efficacy. Notably, there was a significant association between decreased level of gut microbial metabolite butyrate and increased resistance to sorafenib (67). Recent research has initiated an investigation into dietary interventions designed to restore butyrate levels, to assess the potential efficacy of this intervention in improving drug resistance (68). The lack of butyrate-producing bacteria (such as *Butyricicoccus pullicaecorum* and *Roseburia intestinalis*) may serve as an indicator of resistance in patients with HCC undergoing sorafenib therapy. However, further conclusive research is necessary to substantiate this hypothesis.

Resistance remains a significant challenge in the treatment of liver cancer, prompting researchers to investigate various strategies to overcome this obstacle and enhance therapeutic outcomes. Recent studies have substantiated that a reduction in gut microbiota diversity is significantly correlated with resistance to anti-PD-1 therapy. The underlying mechanism may involve a decrease in butyrate-producing bacteria, which impairs CD8+ T cell function. Concurrently, an increase in pathogenic bacteria, such as *Escherichia coli*, may contribute to T cell exhaustion via the LPS-TLR4 signaling pathway (69, 70). Numerous tumor cells circumvent immune system attacks by downregulating the expression of Major Histocompatibility Complex Class I (MHC-I). Studies have demonstrated that specific interventions, including Fhit gene transfection, can restore or augment MHC-I expression, thereby enhancing the efficacy of immunotherapy (71). There is a paucity of research to substantiate the use of microbiota as a supplementary intervention to enhance the efficacy of immunotherapy. The potential for probiotics to indirectly modulate MHC-I expression in tumor cells through the regulation of interactions between gut microbiota and the immune system warrants further investigation, as this may offer a potential approach to improving resistance to sorafenib. Further clinical studies are required to validate the impact of gut microbiota interventions, such as butyrate supplementation, on drug resistance in patients with HCC.

### 4.3 Synergistic treatment model targeting microbiota

Recent advancements in liver cancer treatment research have increasingly focused on the interplay between the microbiome and local immune responses, marking a shift towards precision medicine. Microbiota-targeted therapies have demonstrated significant potential, as the gut microbiota composition in liver cancer patients is closely associated with their clinical characteristics. Personalized interventions may therefore enhance therapeutic efficacy. Current research methodologies include the use of probiotics, prebiotics, synbiotics, and fecal microbiota transplantation (FMT). It is recommended to integrate microbiota modulation strategies to enhance treatment efficacy and minimize adverse effects. Despite the promising prospects, challenges such as individual variability, long-term stability, and tolerance persist, highlighting the need for standardized and personalized approaches in clinical trials.

Inulin, a prebiotic, demonstrates significant potential in tumor immunotherapy, particularly in conjunction with the oncolytic virus T-VEC (72). T-VEC, derived from the herpes simplex virus, has received FDA approval for the treatment of unresectable melanoma. Research indicates that inulin may promote the proliferation of beneficial microbiota, enhance anti-tumor immune responses, and potentially improve gut barrier function, thereby reducing chronic inflammation and creating a more conducive microenvironment for T-VEC. Alterations in the gut microbiota of liver cancer patients have been associated with liver tumor development, and optimizing this microbiota is anticipated to augment the therapeutic efficacy of T-VEC. However, excessive consumption of inulin may elevate the risk of NAFLD and potentially hepatic carcinoma (73). As a prebiotic, inulin plays a role in modulating the gut microbiota and enhancing the production of SCFAs. Nonetheless, an overabundance of SCFAs may disrupt hepatic lipid metabolism, potentially leading to the development of fatty liver. The risks associated with excessive inulin intake warrant further investigation and validation. It is imperative for clinicians and nutritionists to carefully consider individual variability and consumption levels when recommending inulin as a dietary supplement to mitigate potential health risks. Future research should aim to elucidate the dose-response relationship between inulin intake and liver health, thereby providing more robust scientific guidance.

FMT can enhance the effectiveness of immune checkpoint inhibitors and oncolytic virus therapy in treating tumors. Studies indicate that FMT boosts beneficial microbiota, improving patient responses to these treatments and leading to better outcomes. When combined with anti-CTLA-4 antibodies, FMT can synergistically modulate the microbiota and immune system, achieving an objective response rate of 54% (74). FMT can be timed with immunotherapy to optimize gut microbiota, boost the immune system, and improve treatment outcomes. It also enhances oncolytic virus replication in tumors, increasing anti-tumor effects. Studies in mice show that FMT leads to greater tumor shrinkage and longer survival after oncolytic virus treatment, supporting its use in liver cancer. Additionally, FMT combined with oncolytic virus therapy alters gut microbiota, increases beneficial bacteria, and enhances immune status, potentially affecting tumor growth via the gut-brain or gut-immune axis.

Engineered bacteria are gaining attention in liver cancer treatment by using genetic engineering to release immune molecules at tumor sites, enhancing immune responses. For instance, *Escherichia coli Nissle 1917* (EcN) can secrete CXCL16 (75), activating the p38 MAPK pathway to recruit and enhance NK cell activity, showing promise in therapy. Similarly, attenuated *Salmonella typhimurium* can deliver IL-12 to activate CD103+ dendritic cells, boosting immune responses (76). IL-12 enhances T cell and NK cell activation, boosting tumor cell destruction. As liver cancer treatment moves towards immunotherapy, CAR-T cell therapy offers new hope. Researchers are using engineered bacteria to deliver IL-15 and FLT3L at tumor sites, improving CAR-T cell infiltration and local immune response.

However, challenges like microbiota differences, FMT standardization, and safety concerns remain. For example, IL-15 engineered bacteria therapy has a 28% incidence of cytokine release syndrome (77), and severe infections after FMT occur in about 3.5% of cases. Future research must address these issues to optimize FMT and immunotherapy.

## 5 Insights from microbiota-immune crosstalk in HCC associated hepatic disorders

### 5.1 Gut microbiota’s role in liver cancer in NAFLD

NAFLD, a prevalent liver disease globally, is strongly associated with HCC, with gut microbiota imbalance being a major factor. Research indicates notable shifts in NAFLD patients’ gut microbiota, including reduced diversity (Shannon index from 3.8 to 2.9) and increased levels of *Proteobacteria, Escherichia Shigella* and *Erysipelotrichaceae*. The abundance of *Prevotella* in the NAFLD group, as well as the abundance of *Bacteroidetes*, is lower compared to the healthy control group (78). Moreover, elevated fecal counts of Escherichia coli, have been correlated with the occurrence of HCC in patients with cirrhosis (79). In NAFLD patients with liver cancer, fluctuations in gut bacteria are linked to systemic inflammation, indicating a joint role in liver cancer development. Alterations in gut microbiota metabolites like TMAO and the gut virome are also tied to NAFLD severity and may affect its progression to HCC. Modifying gut microbiota could potentially improve NAFLD and lower HCC risk, though the exact mechanisms remain unclear. Further research is essential to uncover liver cancer pathogenesis and enhance early diagnosis and intervention.

### 5.2 Microbial regulation in chronic viral hepatitis

Individuals with chronic viral hepatitis, such as hepatitis B and C, exhibit reduced gut microbial diversity compared to healthy individuals. Specific gut bacterial taxa are associated with liver inflammation, potentially leading to increased fibrosis and a heightened risk of liver cancer. A notable reduction in the *Bifidobacteria*/*Enterobacteriaceae* (B/E) ratio was observed across various stages of liver disease progression, with the most pronounced decrease occurring in patients with decompensated HBV cirrhosis (80). These findings are supported by another study, which demonstrated that HBV infection is associated with an increase in potentially pathogenic bacteria, such as *Enterobacteriaceae*, potentially contributing to the progression of liver disease (81). Additionally, abnormal serum bile acid profiles, characterized by a 2.8-fold increase in glycochenodeoxycholic acid (GCDCA), correlate with reduced hepatic expression of CXCL16 (r = -0.71) (81). In the context of chronic hepatitis B infection, dysbiosis of the gut microbiota may influence viral replication and immune responses. Studies suggest that patients who have achieved a functional cure exhibit a greater abundance of SCFA-producing bacteria in their gut. Notably, butyrate, a specific SCFA, has been shown to inhibit HBV production, indicating that gut microbiota may modulate HBV replication through SCFA-mediated mechanisms. In patients with chronic hepatitis C, microbial translocation and T cell activation are linked to the progression of the disease. Antiviral therapy has been shown to decrease the levels of microbial translocation markers and ameliorate liver damage. Additionally, the association between chronic viral hepatitis and metabolic syndrome underscores the potential involvement of gut microbiota in this process. Research indicates that individuals with chronic hepatitis C frequently exhibit fatty liver, and alterations in the gut microbiota composition may be associated with hepatic fat accumulation (82). This accumulation of fat exacerbates inflammation and fibrosis within the liver, thereby perpetuating a detrimental cycle.

Gut microbiota interventions have potential in enhancing liver function and reducing inflammation in chronic viral hepatitis patients. These therapies can rebalance gut bacteria and boost antiviral treatment effectiveness by strengthening the immune response. Understanding gut bacteria’s role is crucial for preventing and treating liver cancer.

### 5.3 Gut microbiota and liver cancer in AIH patients

AIH patients often experience gut microbiota dysbiosis, potentially linked to liver cancer. In AIH mouse models, impaired gut barriers allow bacteria to reach the liver, worsening inflammation and possibly leading to cancer (83). Both AIH patients and models show gut microbiota changes affecting T follicular helper and regulatory cells (84), disrupting immune detection of liver cancer. Metabolic issues like fatty liver and fibrosis, linked to dysbiosis, are also cancer risk factors. Thus, targeting gut microbiota with probiotics or dietary changes might reduce liver cancer risk in AIH patients.

### 5.4 The oral-gut-liver axis and its immune regulatory role

The oral-gut-liver axis plays a crucial role in metabolism and immune regulation. Alterations in oral microbiota can increase gut permeability, leading to systemic inflammation, metabolic disorders, and liver damage. Harmful oral bacteria can reach the liver via the gut, potentially causing conditions like NAFLD, liver fibrosis (85), and increasing liver cancer risk. Balancing oral microbiota with probiotics and mouthwash, along with dietary changes and supplements, may enhance liver function by regulating this axis.

**Figure 1:**
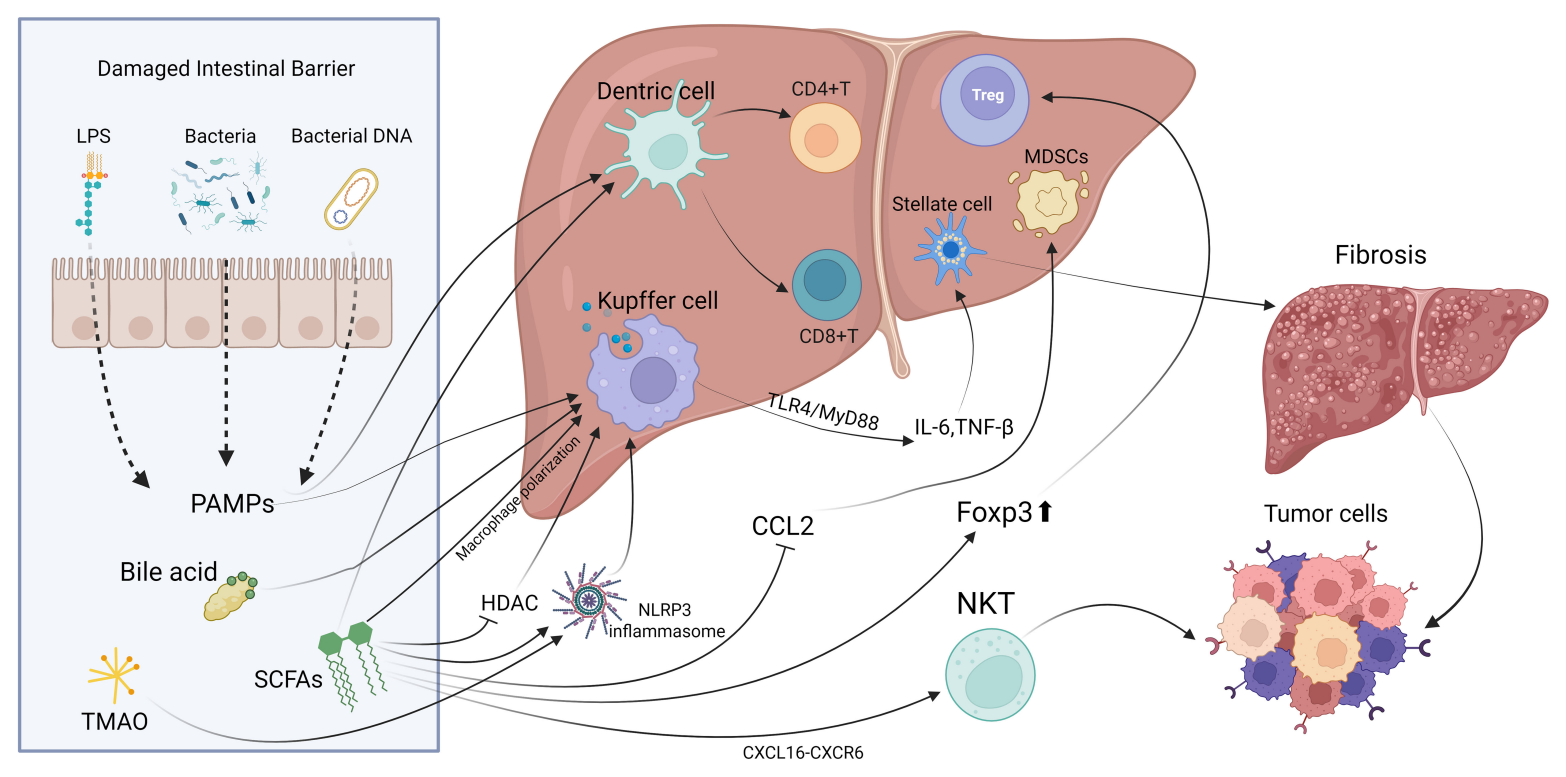
The principal mechanisms and pathways through which gut microbiota interact with local immune cells in the liver.

This review examines the interactions between gut microbiota and the immune environment in liver cancer, highlighting clinical translation prospects. Research shows that gut microbiota influences liver immune balance through metabolic products and molecular patterns via the portal vein, with TLR4/NF-κB and bile acid-FXR/TGR5 pathways being crucial (**Figure 1**). Dysbiosis, like increased Fusobacterium nucleatum and decreased butyrate-producing bacteria, can promote immune evasion in liver cancer by affecting Kupffer cell polarization, T cell balance, and CD8+ T cell exhaustion. Additionally, specific microbiota traits, such as *Akkermansia muciniphila* abundance, may indicate immune therapy response (**Table 1**). Microbiota interventions, including fecal transplants, engineered bacteria, and metabolite supplements, could improve current therapies but are challenged by individual variability and safety concerns. Microbial metabolites like butyrate have concentration-dependent effects, boosting antitumor immunity at low levels and causing tolerance at high levels. More evidence is needed to establish causation, requiring further animal model studies for precise control.

**Table 1:** Microbial community involved in liver cancer immune regulation.

Microbial community	Potential immune regulatory mechanisms	Effect	Ref.
*Akkermansia muciniphila*	Facilitate the activation of CD8+ T cells and modulate their recruitment via its outer membrane protein	Enhance the efficacy of anti-PD-1 therapy and improve the response rate to immunotherapeutic intervention	(66)
*Fusobacterium nucleatum*	Upregulate PD-L1 through the FadA/β-catenin pathway; increase the proportion of Treg; induce epithelial-mesenchymal transition	Facilitate immune evasion, enhance the invasion and metastasis ability of cancer cells and exhibit a positive correlation with the progression of liver metastatic cancer	(86, 87)
*Butyricicoccus*	Enhance CD8+ T cell mitochondrial function via the SCFAs-GPR43 signaling pathway and inhibit HDAC3	Overcoming resistance to immunotherapy and enhancing survival duration	(88, 89)
*Lactobacillus*	Maintain intestinal barrier integrity and reduce LPS translocation	Mitigate TLR4/NF-κB-mediated inflammation and decelerate the progression of liver cirrhosis	(90, 91)
*Bifidobacterium*	Regulate Treg/Th17 balance; increase IL-10 secretion	Inhibit excessive inflammatory response, but may weaken anti-tumor immunity	(92–94)
*Enterobacteriaceae*	Produce TMAO and activate the NLRP3 inflammasome; inhibit CXCL-dependent NKT cell recruitment	Promote the transformation of chronic hepatitis to HCC	(95, 96)
*Prevotella*	Promote IL-6 secretion through TLR4, activate hepatic stellate cells	Facilitate hepatic steatosis, its abundance is associated with the progression of unresectable HCC	(97, 98)
*Escherichia coli*	Produce enterotoxins to disrupt tight junctions; activate Kupffer cells TLR4 pathway.	Increase the risk of HCC in patients with liver cirrhosis	(99, 100)
*Bacteroides*	Regulate bile acid metabolism; inhibit CXCR6+ NKT cell function	May be associated with immune tolerance in HBV related HCC	(101–103)
*Enterococcus faecium*	Inducing ferroptosis by expanding the IFN-γ+CD8+ T cell population	Augment the immunological response against neoplastic cells	(104)

Future research should aim to identify crucial strains, metabolic products, and signaling pathways, understand the origins and dynamics of tumor-associated microorganisms, validate microbial biomarkers, and create personalized interventions like engineered bacteria or metabolic antagonists to boost synergy with current therapies. Technological advances, such as humanized organoid models and AI-driven multi-omics integration, will address microbial diversity and translational challenges, facilitating the transition from mechanistic studies to precise clinical applications and offering new strategies to overcome immune therapy resistance in liver cancer.

## 6 Conclusion

In conclusion, the gut microbiota and its metabolites are vital in liver cancer development, affecting the local immune environment through complex mechanisms. Understanding their interaction with liver cancer enhances our knowledge of its pathogenesis and offers new precision treatment options. Probiotics and microbial management strategies show promise in improving immune responses and influencing disease progression by regulating gut microbiota. Future multi-omics approaches will further advance personalized immunotherapy for liver cancer. Ongoing research and clinical implementation are crucial for the early diagnosis and accurate treatment of HCC, thereby enhancing patient outcomes.
